# Mesenchymal Stem Cell-Derived Exosomes as an Emerging Paradigm for Regenerative Therapy and Nano-Medicine: A Comprehensive Review

**DOI:** 10.3390/life11080784

**Published:** 2021-08-03

**Authors:** Biswajit Panda, Yashvi Sharma, Suchi Gupta, Sujata Mohanty

**Affiliations:** DBT-Centre of Excellence for Stem Cell Research, Stem Cell Facility, All India Institute of Medical Sciences, New Delhi 110029, India; pandabiswajit22@gmail.com (B.P.); yashvi2707@gmail.com (Y.S.); gupta.s1291@gmail.com (S.G.)

**Keywords:** exosomes, mesenchymal stem cells, drug delivery system, therapeutics, nano-medicine, immunomodulation

## Abstract

Mesenchymal Stem Cells are potent therapeutic candidates in the field of regenerative medicine, owing to their immunomodulatory and differentiation potential. However, several complications come with their translational application like viability, duration, and degree of expansion, long-term storage, and high maintenance cost. Therefore, drawbacks of cell-based therapy can be overcome by a novel therapeutic modality emerging in translational research and application, i.e., exosomes. These small vesicles derived from mesenchymal stem cells are emerging as new avenues in the field of nano-medicine. These nano-vesicles have caught the attention of researchers with their potency as regenerative medicine both in nanotherapeutics and drug delivery systems. In this review, we discuss the current knowledge in the biology and handling of exosomes, with their limitations and future applications. Additionally, we highlight current perspectives that primarily focus on their effect on various diseases and their potential as a drug delivery vehicle.

## 1. Introduction

Mesenchymal stem cells (MSCs) have established their reputation as therapeutically beneficial agents owing to their immunomodulatory and immunosuppressive features. They have immense regenerative capabilities and can be derived from multiple tissue sources, including bone marrow, Wharton’s jelly, adipose tissue, dental pulp, etc. The potential of MSCs has been maximized in the alleviation and prevention of a plethora of diseases. Studies related to the fate of MSC injections in vivo indicate that these cells migrate to, and get caught up in, the lung vasculature primarily and yet can showcase their healing capabilities at distant sites; this hints about the probable paracrine mechanism of action for these stem cells. Eventually, it was found that MSCs exert their therapeutic effects by releasing extracellular vesicles, thereby mediating their functionality and initiating contact with diseased cells. A subset of these extracellular vesicles in the 30–150 nm size range, known as exosomes, has gained extreme importance in recent times. Exosomes were previously considered the ‘dust’ or ‘garbage bags’ of cells but have recently stolen the limelight as an agent mediating intercellular contacts by virtue of their small size, ubiquitous secretion, omnipresence, and ease of their migration in vivo. The biogenesis of exosomes is of great interest because of their endosomal origin; this occurs via the classical ESCRT dependent pathway and the noncanonical pathway mediated by Alix and syntenin. Therapeutic focus is thereby shifted from MSCs toward these nanovesicles due to the limitations possessed by MSCs, including viability, duration, and degree of cell expansion, long-term storage incurring the high cost of maintenance, etc. These nanosized particles carry an intricate range of molecules, are secreted by all cells, and are present in all biological fluids, which potentially establishes them as a suitable candidate for drug delivery and diagnostic purposes. As a result of these features, exosomes are also being considered for downstream clinical applications because they exhibit the MSC characteristics, including an immunomodulatory nature, immune suppression, low immunogenicity, and oncogenicity. MSC-derived exosomes have been applied in a wide spectrum of diseases, such as cardiovascular disease, liver disease, neurological disorders, kidney diseases, etc. Recently, the COVID-19 pandemic brought MSC-derived exosomes to the limelight for their regenerative and healing abilities. However, research in the field of exosomes is still nascent in terms of understanding its mechanics, molecular workforce, and standardized handling. This review holistically discusses the biology of exosomes, their potential as a drug delivery vehicle, and therapeutic applications.

## 2. A Biological and Mechanistic Approach to Confer the Potential of Exosomes: A General Account

### 2.1. Biogenesis of Exosomes

**Figure 1:**
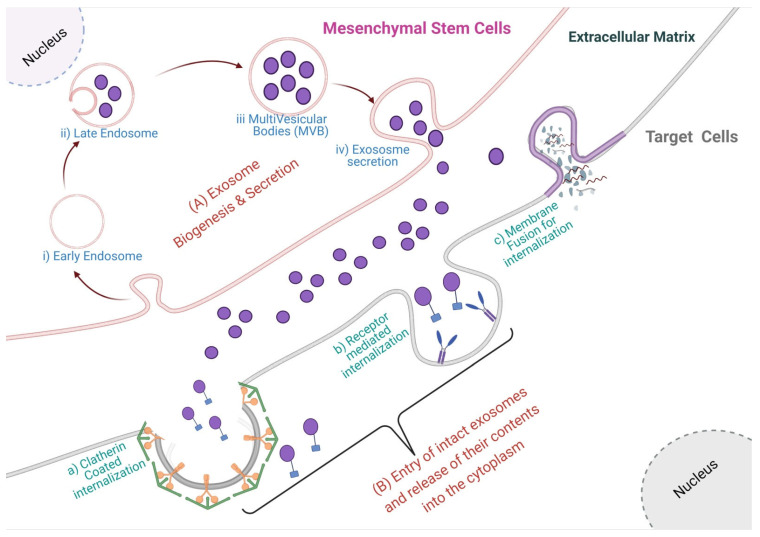
(**A**) Biogenesis, secretion, and cellular uptake of exosomes. The biogenesis of exosomes starts from the (i) early endosomes which mature into (ii) late endosome, then (iii) multivesicular bodies are formed by the invagination of late endosomal membrane, which is finally secreted as (iv) exosomes to the extracellular matrix in a mesenchymal stem cell. (**B**) The secreted exosomes are uptaken by a recipient cell in several ways viz. (a) clathrin-mediated uptake, (b) receptor-mediated uptake or by the (c) membrane fusion event.

Exosomes are nanosized membrane vesicles with sizes ranging from ~40–160 nm, originating from the endosomal pathway [1,2,3]. The late endosomal limiting membrane invaginates into multivesicular bodies (MVBs) containing intraluminal vesicles (ILVs). ILVs are ultimately secreted as exosomes through the MVB fusion to the plasma membrane and exocytosis [1,4,5]. Several studies have reported the importance of ESCRT machinery in this process [2,6,7,8,9]. This complex is composed of ~30 types of proteins assembled into four distinct complexes (numbered from ESCRT 0 to III) with some associated proteins (VPS4, VTA1, ALIX) [6]. ESCRT 0 recognizes and sequesters ubiquitinated proteins in the endosomal membrane and recruits ESCRT I and II [10]. Ubiquitin (Ub) acts as a signal for exosomal cargo sorting on the endosome membrane. Then, ESCRT I and II initiate intraluminal membrane budding by binding to the outer surface of the endosomal membrane near the ubiquitinated protein cargos, thereby selecting them to be in the newly-formed intraluminal buds in the MVB and serving an important role in cargo sorting. ESCRT III completes the process by sequestrating MVB proteins. After ILVs are generated, ESCRT III is separated from the MVB membrane by the sorting protein VPS4 [11]. However, some evidence shows that silencing key genes involved in the ESCRT pathway does not inhibit MVB formation, suggesting the existence of an ESCRT-independent pathway [12]. For example, the ubiquitous transmembrane proteins, syndecans (SDC1-4), directly regulate the ILVs during exosome formation by coaccumulating with syntenin and ALIX in exosomes [13]. Additionally, the role of lipids in exosome biogenesis has also been reported by finding that sphingolipid ceramide is required for ILV formation. Neutral sphingomyelinase (nSMase) facilitates ILV formation by promoting MVB budding. In this pathway, exosomes are enriched with proteolipoprotein, CD63, CD81, and TSG101 [14] (Figure 1).

### 2.2. Exosome Secretion and Internalization

The release of exosomes into the extracellular milieu is governed by an orchestration of proteins viz. soluble *N*-ethylmaleimide- sensitive factor attachment protein receptors (SNAREs), tethering factors, Rabs, and other Ras GTPases [15]. The SNARE proteins, R- or Q-SNAREs, have been reported to affect exosome release. Fader et al. showed that the R-SNARE vesicle-associated membrane protein 7 (VAMP7) is necessary for exosome release in the human leukemic cell line K562 [16]. Another R-SNARE protein, YKT6, is required for exosome release, as shown by two independent studies. Gross et al. showed that depletion of YKT6 decreased the level of TSG101, WNT3A, and VPS26/35 in exosomes secreted from human embryonic kidney HEK293 cells [17]. Further, Ruiz-Martinez et al. showed a reduced level of exosome-associated TSG101 after the knockdown of YKT6 in A549 human lung cancer cells [18]. Similarly, in *Drosophila* S2 cells, depletion of the Q-SNARE syntaxin 1A (Syx1A) decreased the release of EV enriched v exosomes [19]. Wei et al. reported that pyruvate kinase type M2 (PKM2) phosphorylates SNAP-23, thus enabling exosome release [20]. Although most studies on the molecular mechanism of exosome release are on cancer, few (almost none) have reported on mesenchymal stem cell exosomes [21,22].

Rab GTPases, the largest family of small GTPases, regulate many steps of membrane trafficking, including vesicle budding, transport of vesicles along actin and tubulin, and membrane fusion [23], are also involved in exosome secretion. Several studies demonstrated that Rab family proteins (Rab2b, Rab5a, Rab27a, Rab27b, Rab35, and Rab11) are involved in this process [24]. Additionally, it has also been shown by Yu et al. that the tumor suppressor protein p53 may also influence exosome secretion through regulating transcription genes such as TSAP6 and CHMP4C [25]. Apart from that, various stimuli and changes like cell membrane pH and the concentration of K+ may also trigger the secretion of exosomes [26,27].

### 2.3. Isolation of Exosomes: The First Step towards Pharmaceuticalization

**Figure 2:**
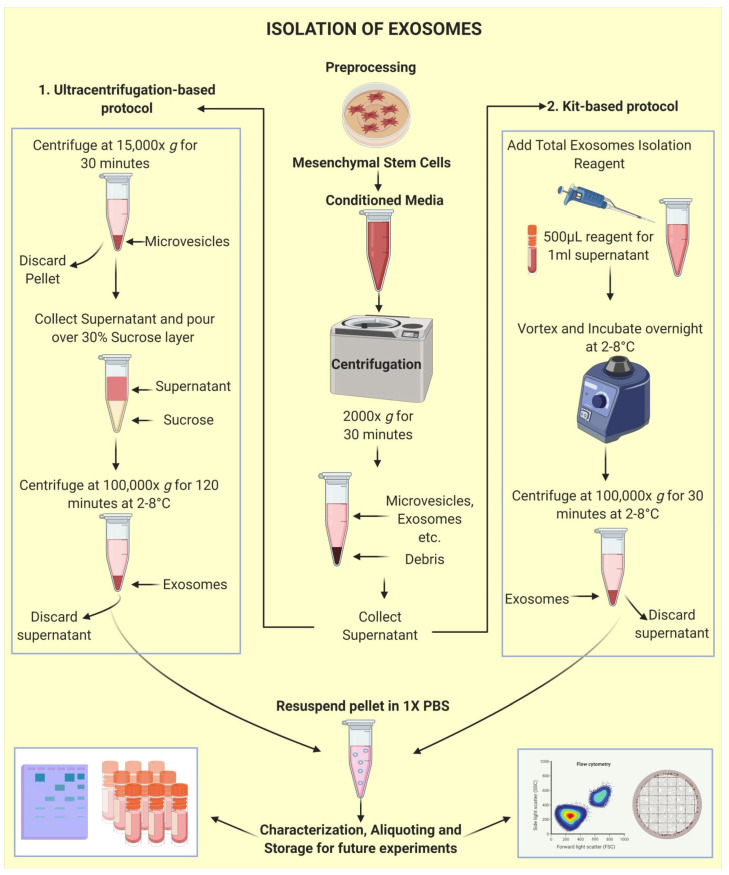
Isolation of Exosomes: exosomes are commonly isolated from the conditioned media. Some common preprocessing steps are required for both the techniques, including collecting conditioned media from MSCs, performing a centrifugation round at 2000× *g* for 30 min to remove debris. Furthermore, the conditioned media can be subject to any of the two techniques including, Ultracentrifugation (1) or Kit-based methods (2) for isolation of exosomes. These exosomes can be further used for characterization, aliquoting, and storage for future experiments.

MSC-derived exosomes are being considered a novel tool for cell-free therapeutics [28,29,30,31]; however, the cardinal step in evaluating the extent of their competence is to successfully isolate and purify exosomes and obtain a good yield. Although a great deal of experimentation has been performed, there is still no uniformity in isolation methods; but, by far, the technique considered best is “ultracentrifugation” due to the superlative quality of exosomes isolated within it and the ubiquity of its use [32,33]. Basic ultracentrifugation as an exosome isolation technique was introduced by Johnstone et al. [34] to infer that vesicle shedding was an intermediate process during maturation to erythrocytes. There have been several advancements to this process, such as modulation in the number of cycles of centrifugation [35] and optimization in protocols of differential ultracentrifugation [36,37], density gradient ultracentrifugation [32,38,39,40], etc. Certain isolation kits have also been devised to be considered a time-saving alternative showing reasonable results [41,42,43]. The possibility of combining the beneficial effects of ultracentrifugation and precipitation-based kits was explored by Ryu et al. [44]. They inferred that combining the potential of both techniques was expedient for the isolation of small EVs, provided a good output, and held no lags about their constitution, hence utilizable for catering to massive sample-based critical clinical evaluations. Common protocols used for exosome isolation are shown in Figure 2.

Despite abounding attempts to find a robust technique for uniform, use globally, many shortcomings exist that need to be addressed, such as long duration, complicated protocols, need for special equipment, lack of cost-effectiveness, limited utility, the requirement of large volumes of sample, lack of specificity, truncated yield, low rate of recovery, dubious purity, and risk of mechanical damage. These techniques, in their current form, are not suitable for standardization. All the techniques have their advantages and drawbacks; however, a technique that could satisfactorily channel the benefits of all pre-existing technologies collectively while facilitating exosome isolation for downstream processing at a translational level to visualize the use of exosomes for future applications like drug formulation and delivery of therapeutics, is yet to be devised.

### 2.4. Characterization and Visualization of Exosomes

In order to entirely comprehend the functional, spatial and temporal properties of exosomes, it is imperative to perceive its characterization, including labeling, imaging, and visualization. As per the ISEV guidelines (2018) for EV characterization [45], EVs should possess at least three positive protein markers, including at least one transmembrane/lipid-bound protein, one cytosolic protein, and at least one negative protein marker, as mentioned in the MISEV (2018) [45]. For characterizing individual vesicles, two different but complementary techniques can be used [45]. Some most popular techniques include regular fluorescence microscopy, SEM, TEM, Cryo-EM, AFM, NTA, Flow Cytometry [45]. Several assays are performed to check the size range and distribution, concentration and spread of exosomes, shape, trajectories and particle velocity, structural features including surface proteins, chemical and physical properties, along with the cargo properties for the isolated extracellular vesicles. Differences in these characteristics, especially in their size, shape, and surface proteins, are checked to differentiate exosomes from other extracellular vesicles like micro-vesicles and apoptotic bodies. 

The basic characterization techniques standardized for the detection of exosomes include nano-particle tracking analysis, which is used to assess the size and yield of the exosomes. Further western blotting and flow cytometry can be used to detect exosome-specific markers. The commonly used markers to identify exosomes in most experiments are tetraspanins (CD9, CD63, CD81), TSG101, syntenin-1, Alix, Hsp90α, Hsp70, LAMP2, cofilin, flotillin-1 [46,47,48,49,50,51]. Apart from identifying the presence of exosomes, this technique is also applied to detect the expression of proteins ferried by exosomes [52,53]. Another prime technique used for the characterization and visualization of exosomes is electron microscopy, which provides comprehensive information about their configuration. However, exosomes via electron microscopy cannot be visualized in their native state due to the pre-treatments required for this technique; furthermore, they appear rather cup-shaped or saucer-shaped instead of their native round shape due to dehydration during sample preparation [54,55,56]. A variation to EM is cryo-electron microscopy [57]. An advantage of this technique is that exosomes can be visualized in their native round forms, thereby avoiding artifacts due to fixation [58]. Extracellular vesicles can also be tracked by optical microscopy within the visible light range (380–750 nm) using bioluminescence labeling or fluorescence labeling by creating fusion proteins even though many alterations have been experimented with in all of these techniques. Yet, no explicit tool or technology can comprehensively describe all the facets of analysis of extracellular vesicles. While there are shortcomings in every technique when used individually, they may be counterweighed by the benefits of another technique if explored in a synergistic manner [59,60].

## 3. The Therapeutic Nature of MSC Derived Exosomes by the Synergistic Functioning of miRNAs and Proteins

MSCs partially function via a paracrine mechanism by the secretion of exosomes. These small vesicles carry a broad range of cargo, which aids its therapeutic and regenerative capabilities in a vast spectrum of diseases and ailments. The therapeutic activities of MSC-derived exosomes are mediated majorly via the horizontal transfer of its cargo components, which then regulate and modulate the behavior of recipient cells by an array of mechanisms [61]. MSC-derived exosomes exert some significant effects specifically by virtue of the proteins and miRNAs they carry, apart from other bioactive molecules like lipids, DNA, long coding mRNA, tRNA, etc. The proteins they carry may exhibit any kind of functional, structural, or enzymatic activities, whereas miRNAs are small noncoding sequences that may result in epigenetic modifications by the mechanism of RNA silencing. These miRNAs can either cleave or destabilize the target mRNAs or result in the modulation of mRNA transcription into protein, probably by reducing its efficacy. The cargo carried by MSC-derived exosomes differs based on tissues from which MSCs were derived [29]. The miRNAs constituted by MSC-derived exosomes can influence various developmental and regulatory processes and also play a role in tumorigenesis and tumor progression. 

The RNA profile of MSC-exosomes derived from porcine adipose tissue was characterized by Eirin et al. [62] using RNA-seq technology. They claimed that vesicles from porcine-derived MSCs preferentially contain discrete mRNAs and miRNAs when contrasted with their parent cells and stated that several mRNAs encoding transcription factors, Golgi proteins, and proteins for TGF-β signaling were found in the cargo of exosomes. These included transcripts for POU3F1 (TST-1, OCT6), JARID2, p53-negative regulators- MDM434 & PEG3, HGF, HES1, TCF4, CEBPA, KF7, GOLGA4, ARRB1, IFT57, TGFB1, FURIN, GAS7, HMGA2, LIN28B, which were involved in a diverse array of processes like stem cell functionality, angiogenesis, splicing, cell death, adipogenesis, proteolysis, and organization of genetic material. All these factors were found to be regulated by the miRNAs, like miR148a, miR532-5p, miR378, let-7f, etc., which are also contained in exosomes. 

It was also stated that cytoskeletal proteins and those related to mitochondrial and calcium signaling were selectively segregated out of the vesicles. Furthermore, several proteins, like EGF, FGF, and PDGF, involved in enhancing angiogenesis were found to be significantly upregulated in MSCs under peripheral arterial disease-like conditions, and it resulted in an enhanced angiogenic signaling profile of the exosomes. Therefore, they are individually capable of inducing angiogenesis [63]. miRNAs are also known to improve conditions related to heart disease. An enhanced expression of miR-29 and miR-24, and downregulation of miRNAs, such as miR-21, miR-15, miR-34, miR-130, and miR-378, can help in the alleviation of heart disorders by different mechanisms, such as limiting the aortic vascular inflammation, inhibiting apoptosis of cardiac muscle cells, reducing the size of infarction, preventing hypertrophy and preventing cardiac dysfunction, thereby also reducing the risk of cardiac ischemic injuries [64]. 

The Vascular Endothelial Growth Factor (VEGF) enriched in these vesicles upregulate the transmembrane ligand for Eph receptor tyrosine kinases (Ephrin-B2) and enhances VEGF-induced angiogenesis, thereby inferring that these vesicles not only deliver the protein VEGF but also upregulate its creation in the receiver cells [65]. This effect can also be accounted for by several miRNAs contained in MSC exosomes involved in inducing angiogenesis and alleviating diseases, including miR-132, miR-125a, miR-1246, miR-23a, etc. Therefore, it can be stated that MSC-derived exosomes not only provide ready therapeutic agents but also mechanisms for their regulation. A detailed proteomic characterization of MSC-exosomes derived from bone marrow, adipose tissue, and umbilical cord revealed 355 proteins common to all sources. It was found that proteins in MSC-exosomes (Table 1) that derived from bone marrow majorly functioned in the activation of granulocytes, in regulating cell migration, and binding protein complexes and integrins. In the case of exosomes derived from adipose tissue and the umbilical cord, the proteins were maximally involved in activation of leukocytes and binding of cell adhesion molecules [66]. The systematic study of miRNAs’ functionality is a very intricate process owing to the diversity of miRNAs, including the pathways and proteins they target. However, bioinformatics tools can help to establish miRNA landscapes that provide an “all-in-one approach” which describe the relationship between miRNAs and their target genes and proteins. These tools may be beneficial for enhancing means to search for miRNAs with inherent therapeutic capabilities to formulate MSC derived exosome-based, off-the-shelf remedial agents [67]. 

## 4. Therapeutic Potential of MSC Derived Exosomes in Various Diseases

**Figure 3:**
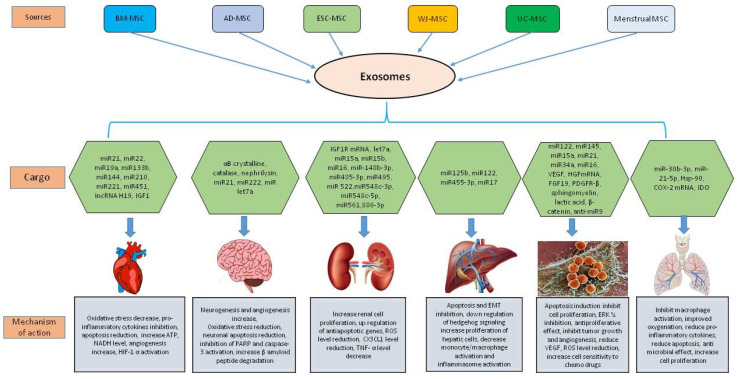
Mesenchymal stem cell exosome cargo in modulating cardiovascular diseases, neurological disorders, kidney diseases, liver diseases, cancer, and lung diseases.

As paracrine effectors, MSC-derived exosomes have gained much attention in the last few years as a promising candidate for cell-free therapeutics in a wide spectrum of pathophysiological conditions. In the following paragraphs, we have shed light on the therapeutic potential of these exosomes in different diseases (Figure 3, Table 2).

### 4.1. MSC Derived Exosomes in Cardiovascular Diseases

Several preclinical studies have demonstrated the efficacy of MSC-derived exosomes for CVD treatment. Lai et al. showed that the supernatant of human embryonic stem cell (ESC)-derived MSCs contained small particles (50–100 nm in diameter) corresponding to exosomes. When administered to a mouse myocardial ischemia/reperfusion injury model, these exosomes remarkably reduced the infarct size [68]. The authors also administered exosomes secreted from human ESC-derived MSCs to a mouse model of AMI (Acute Myocardial Infarction), showing a reduced infarct size and improved cardiac function [69]. Furthermore, they demonstrated that the phosphorylation of Akt and GSK3 (possessing anti-apoptotic effects) significantly increased, and that c-jun N-terminal kinase (possessing proapoptotic effects) significantly decreased in cardiac tissue following exosome administration. Bian et al. collected extracellular vehicles (EVs) from hypoxic human BMMSCs and administered the EVs to a rat AMI model. The study showed that EV administration significantly reduced infarct size, restored cardiac function, and stimulated angiogenesis in the ischemic zone [70]. Feng et al. demonstrated that exosomes secreted from mouse BMMSCs after ischemic preconditioning contained a greater amount of miR-22 [71]. When administered to mice with AMI, these miR-22-enriched exosomes significantly reduced infarct size and cardiac fibrosis, possibly through the downregulation of methyl-CpG-binding protein 2. In another study, Yu et al. used MSCs overexpressing the transcription factor GATA-4 (MSC_GATA-4) and demonstrated that the administration of MSC_GATA-4-derived exosomes restored cardiac function and reduced infarct size in a rat model of AMI. The study also showed that MSC_GATA-4-derived exosomes expressed a greater amount of miRNAs, particularly miR-19a, which appeared to be involved in the cardioprotective effect of MSC_GATA-4-derived exosomes via the downregulation of phosphatase and tensin homolog (PTEN) and subsequent activation of anti-apoptotic Akt [72]. Similar cardioprotective roles of MSC exosomes were also shown by Wang et al. using endometrium-derived MSCs (EnMSCs). Their study suggested that miR-21 contained in EnMSC-derived exosomes mediated cardioprotective effects via the downregulation of PTEN and subsequent activation of Akt, resulting in the upregulation of Bcl-2 and vascular endothelial growth factor [73].

Recently, Huang et al. showed that the therapeutic efficacy of MSC-derived exosomes in AMI can be enhanced by atorvastatin (ATV), one of the most widely used lipid-lowering drugs for patients with coronary heart disease. The authors showed that exosomes derived from ATV-pretreated MSCs (MSC^ATV^-Exo) significantly improved cardiac function and promoted blood vessel formation compared with exosomes derived from non-pretreated MSCs (MSC-Exo) via an increased level of lncRNA H19 expression. [74]. The decrease in apoptosis in H9C2 cardiomyocyte cells by administration of BMMSC derived exosomes enriched in miR-144 was demonstrated by Wen et al. [75]. The exosomes mediate this function by targeting the PTEN/AKT pathway (decreased PTEN expression and increased p-AKT expression), as evident from the study. Similarly, a study by Cheng et al. also shows the efficacy of exosomes in attenuating post-infarction cardiac apoptosis. The authors showed that hypoxia challenged MSC-derived exosomes enriched in miR-210, reduced infarct size, and improved heart function after coronary ligation both in vitro and in vivo stress [76]. Preclinical studies have also reported the beneficial effects of exosome administration on neurological recovery following stroke induction. Xin et al. found that the systemic administration of rat BMMSC-derived exosomes after inducing stroke via ligating the middle cerebral artery significantly enhanced neurological recovery and stimulated neurogenesis and angiogenesis in the ischemic boundary zone [77]. The authors also demonstrated that administration of BMMSCs overexpressing miR-133b (MSCs_miR-133b) in a rat stroke model enhanced the recovery of neurological function. Furthermore, they showed that the expression of connective tissue growth factor (CTGF), a target for miR-133b, was significantly reduced in the ischemic boundary zone after MSCs_miR-133b administration; this suggests that exosome-derived miR-133b was implicated in the MSC-mediated recovery of neurological function in the model. However, Doeppner et al. showed that improvement in neurological function and stimulation of neurogenesis and angiogenesis at the ischemic boundary remained the same in both BMMSC administration and BMMSC-derived EV administration [78].

### 4.2. MSC Derived Exosomes in Neurodegenerative Diseases

The ability of exosomes to cross the BBB (Blood–Brain Barrier) establishes them as a potential candidate for drug delivery to the brain in various neurodegenerative diseases. Several studies support this concept. For instance, it has been suggested that exosomes enter CNS via two mechanisms: uptaken by endothelial cells and crossing into the cell through transcytosis, or crossing intercellular junctions between endothelial cells and entering the CNS [79,80]. It has been shown that exosome-associated miR-105 can downregulate the expression of ZO-1, a critical molecular component of tight junctions, hence demolishing the barrier action of endothelial cells [81]. Exosomes have produced beneficial effects in a variety of models for neurodegenerative diseases, such as Parkinson’s disease. Jarmalavičiūtė et al. reported that exosomes obtained from human dental pulp stem cells could suppress apoptosis of dopaminergic neurons following treatment with 6-OHDA (6-Hydroxydopamine) in a Parkinson’s disease model [82]. 6-OHDA induces apoptosis through the generation of reactive oxygen species (ROS), suggesting that exosomes can decrease the sensitivity of dopaminergic neurons to oxidative stress [83]. Furthermore, it has been shown that chaperone αB crystalline, a pigment produced by the human retinal epithelium, is found inside exosomes, which may play a protective role against oxidative stress in retinal cells [84]. Haney et al. showed that mouse macrophage-derived exosomes remarkably enhanced cell survival against 6-OHDA-induced injury [85]. Interestingly, exosomes caused a reduction in ROS levels in activated macrophages regardless of whether they were loaded with catalase, suggesting that exosomes might function similarly in microglial cells under neuroinflammatory conditions. Furthermore, in vivo studies have suggested that exosomes loaded with catalase causes a decrease in microgliosis and improves the survival of dopaminergic neurons in mice treated with 6-OHDA [86]. Hence, it can be suggested that stem cell-derived exosomes produce neuroprotection through reduced oxidative stress. 

Several studies have suggested a neurotherapeutic behavior of exosomes obtained from adipose tissue-derived MSCs (ADMSCs). ADMSCs can secrete neprilysin-bound exosomes [87]. As a type II membrane-associated metalloendopeptidase, neprilysin is reportedly a critical proteolysis product in the cleavage of β-amyloid. It was demonstrated that the expression and function of neprilysin were reduced in patients with Alzheimer’s disease [88]. ADMSC-derived exosomes display neprilysin-related enzyme activity and are involved in reducing β- amyloid levels in neuroblastoma cells. Additionally, these exosomes express greater amounts of neprilysin compared with BMMSCs, emphasizing functional and activity differences in exosomes obtained from different sources [89]. It was also indicated that exosomes obtained from murine adipose tissue-derived MSCs improved the survival of human neuroblastoma cells and protected the murine hippocampal neurons from oxidative damage [90]. Furthermore, the authors of this study showed that exosomes obtained from murine ADMSCs enhanced remyelination and stimulated the progression of oligodendroglial progenitors [90]. These neuroprotective effects of exosomes are also supported by Bonafede et al. in an in vitro model of ALS (Amyotrophic lateral sclerosis). The study showed that administration of murine ADMSC-derived exosomes in a motor-neuron-like cell line expressing a high amount of SOD1, hence under oxidative stress, protects the motor-neuron-like cells from oxidative injury [91]. These results highlight the potential of applying MSC-derived exosomes as a therapeutic tool to treat motor neuron disorders. 

### 4.3. MSC Derived Exosomes in Kidney Diseases

Recent studies on the therapeutic potentials of MSC-derived EVs suggest their ability to regenerate injured renal cells in experimental acute kidney injury (AKI) and chronic kidney disease (CKD) models. Results from these studies suggest that EVs exert their trophic and reparative effects by shuttling their cargo of genes, microRNAs, and proteins to recipient cells in the kidney, attenuating renal injury and improving its recovery competence. In an in vitro model of cisplatin-induced AKI, Tomasoni et al. demonstrated that coincubation of damaged proximal renal tubular epithelial cells with MSC-derived EVs, which are selectively enriched with IGF1R mRNA, enhanced cell proliferation and repair, suggesting that the transfer of this gene to tubular cells is an important mechanism by which MSCs confer renoprotective effects in experimental AKI [92]. Bruno et al. reported that human adult MSC-derived microvesicles, which include exosomes, mimicked the protection against AKI as provided by intravenously administered MSC [93]. RNase treatment of EVs abrogated EV-induced in vitro proliferation and resistance to apoptosis, indicating that the mRNAs shuttled by EVs activate a transcriptional program of repair in recipient cells. In line with this observation, EVs released from kidney-derived MSCs preincubated with RNase failed to ameliorate TGF-β1-induced peritubular capillary rarefaction and tubulointerstitial fibrosis in mice with unilateral ureteral obstruction (UUO) [94]. Bruno et al. further showed that a single intravenous administration of MSC-derived EVs improved mouse survival after injecting a lethal dose of cisplatin, whereas multiple EV injections further decreased mortality and preserved renal structure and function [95]. Administration of MSC-derived EVs upregulated the expression of the anti-apoptotic genes BCLX, BCL2, and BIRC8, but downregulated the expression of the pro-apoptotic genes CASP1, CASP8, and LTA in cisplatin-treated human tubular epithelial cells, suggesting that modulation of apoptosis may contribute to MSC-derived EV-induced renal repair. Using an in vitro model of ischemia-reperfusion injury (IRI) induced by ATP depletion in renal proximal tubular epithelial cells, Lindoso et al. found that incorporation of MSC-EVs in damaged cells modulated several microRNAs related to important processes in renal recovery [96]. Renal oxidative stress and inflammation are also known to be modulated by MSC-derived EVs. Renal expression of the NADPH oxidase (NOX)-2 is upregulated in rats with IRI but not in those treated with intravenous MSC-derived EVs [97]. Interestingly, this intervention not only alleviates oxidative stress but also reduces apoptosis and enhances renal cell proliferation, suggesting that post-transcriptional regulation of NOX2 in renal recipient cells may be implicated in MSC-derived EVs-induced renal repair. In rats with IRI, MSC-derived EVs alleviated renal inflammation and improved renal function by suppressing the expression of C-X3-C motif ligand-1 (CX3CL1), a potent chemo-attractant protein for macrophages that also promotes interstitial fibrosis [98]. Interestingly, MSC-derived EVs were enriched with miR-16, miR-15b, and miR-15a, all of which target CX3CL1, suggesting that post-transcriptional modulation of CX3CL1 is an important mechanism by which MSC-derived EVs mitigate inflammation and renal injury in ischemic AKI. Promising results from these experimental studies provided the impetus to apply MSC-derived EVs to address the clinical needs of patients with renal disease. However, only a few clinical trials investigated the safety and therapeutic efficacy of MSC-derived EVs in patients with kidney diseases. Nassar et al. published their results of a phase II/III clinical trial using cord tissue MSC-derived EVs to ameliorate the progression of chronic kidney disease (CKD) [99]. In this study, 20 patients who had been diagnosed for more than 6 months with chronic kidney disease (eGFR 15–60 mg/mL) were treated with two doses (1 week apart) of MSC-derived EVs (100 μg/kg/dose). Patients treated with MSC-derived EVs exhibited improved eGFRs and urinary albumin creatinine ratio, as well as significant decreases in BUN and creatinine after 1 year. In addition, the patients showed a significant increase in plasma levels of TGF-B and IL-10 with persistent, significant decreases in TNF-α. Similarly, Ingato et al. showed their results from a single-center, randomized, placebo-controlled, phase II/III clinical pilot study that recruited 40 patients with stage III-IV CKD (eGFR between 15–60 mg/mL/min), who were randomized to receive either placebo or two doses (first intravenous and second intraarterial) of MSC-derived EVs, one week apart [100]. After a 12-month follow-up, EV-treated patients exhibited a significant improvement in renal function (improved eGFR and decreased serum creatinine, BUN, and albuminuria). Clinical improvement paralleled changes in plasma levels of several immune-inflammatory markers, including TNF-α, TGF-β1, and IL-10. These observations suggest that MSC-derived EVs are safe and can ameliorate the inflammatory immune reaction and improve the overall kidney function in CKD patients.

### 4.4. MSC Derived Exosomes in Liver Diseases

MSC administration in animal models of liver fibrosis/cirrhosis has been shown to ameliorate the disease [101]. Similar results are also found using the MSC-conditioned media [102], suggesting that MSC might achieve their role in vivo through their secreted exosomes. Using a carbon tetrachloride (CCl_4_)-induced liver injury model in Kunming mice, Li et al. showed that the exosomes derived from human umbilical cord MSCs ameliorate liver fibrosis by inhibiting both the epithelial-mesenchymal transition of hepatocytes and collagen production. The exosomes were found to significantly restore the serum aspartate aminotransferase activity and inactivate the TGF-β1/Smad signaling pathway by decreasing collagen type I/III and TGF-β1 and the phosphorylation of Smad2 [103]. Another study showed that chorionic plate-derived MSCs can release exosomes containing miR-125b, mediate miR-125b transfer between MSCs and target cells, such as Hedgehog (Hh)-responsive hepatic stellate cells (HSCs), and thus alleviate hepatic fibrosis in CCl_4_-treated Sprague–Dawley rats by impeding the activation of Hh signaling via the inhibition of Smo expression [104]. Lou et al. showed that exosomes produced by adipose tissue-derived MSCs (AD-MSC-122) expressing miR-122 were more effective than those expressing scramble miRNA or naive exosomes in reducing the proliferation and activation of the human HSC cell line LX2 or primary HSCs from C57BL/6 mice [105]. AD-MSC-122-derived exosomes could transfer miR-122 into HSCs cells and then regulate the expression of miR-122-target genes, such as P4HA1 and IGF1R, which are involved in the proliferation and collagen maturation of HSCs [106]. These studies indicate that MSCs show their therapeutic efficacy via miR-122 present in the exosomes, thereby representing a new strategy for treating liver fibrosis. The therapeutic effects of MSC-derived exosomes/EVs have been reported in several experimental models of acute kidney, cardiac, and lung injury. However, only a few studies are currently available on the therapeutic effects of MSC exosomes in acute liver injury. Tan et al. found that HuES9.E1 MSC-derived exosomes elicit hepatoprotective effects in both in vitro models of acetaminophen or H_2_O_2_-induced hepatocyte injury and a C57BL/6 mouse model of CCl_4_-induced acute liver injury, through an increase in hepatocyte proliferation, demonstrated by elevated proliferating cell nuclear antigen and high cell viability. The increased survival rate is associated with the upregulation of genes involved in the priming phase liver regeneration, which subsequently leads to high expression of proliferation proteins (proliferating cell nuclear antigen and Cyclin D1), the anti-apoptotic gene *Bcl-xL*, and the signal transducer and activator of transcription 3 (STAT3) [107]. Recently, a study by Shao et al. showed that administration of human umbilical cord mesenchymal stem cells (hUC-MSCs)-derived miR-455-3p-enriched exosomes suppressed monocyte/macrophage activation and alleviated acute liver injury by inhibiting IL-6 signaling (by targeting the PIK3r1 gene) in a carbon tetrachloride (CCl_4_)-induced liver injury in a mice model [108]. Furthermore, a study on a lipopolysaccharide/d-galactosamine-induced acute liver failure mice model by Liu et al. showed that adipose tissue-derived MSC (AMSC) exosomes alleviate acute liver failure (ALF) by reducing serum alanine aminotransferase and aspartate aminotransferase levels and hepatic inflammasome activation. The miR-17, which can suppress NLRP3 inflammasome activation by targeting TXNIP expression, is abundant in AMSC-Exo cargo; this clearly indicates that AMSC-Exo-based therapy may be a promising approach for treating TXNIP/NLRP3 inflammasome-related inflammatory liver diseases [109]. 

### 4.5. MSC Derived Exosomes in Cancer

The role of MSCs in cancer is a debatable topic. This area of cancer research, in the light of exosomes, has been gaining momentum in the past few years. Several studies have shown that MSCs act as a double-edged sword in both tumor suppression, or progression in different tumor models [110,111,112,113]. However, the mechanisms remain elusive. The EVs secreted by MSCs contain paracrine factors through which they mediate their effects on tumor progression [114]. For instance, exosomes released from multiple myeloma patient BMMSCs promote multiple myeloma tumor growth in SCID-beige mice. BMMSC derived exosomes promote gastric or colon tumor growth in BALB/c nu/nu mice by enhancing the expression of vascular endothelial growth factor (VEGF) in tumor cells [115,116]. They facilitate nasopharyngeal carcinoma progression and migration in non-obese diabetic/severe combined immunodeficient (NOD/SCID) mice by activating the FGF19-FGFR4-dependent ERK signaling cascade and epithelial-mesenchymal transition [117]. Vallabhaneni et al. demonstrate that the exosomes secreted from hMSCs are rich in miR-21 and 34a, supporting breast cancer cell proliferation and metastasis [118]. 

However, antitumor effects are also exhibited by exosomes [119]. Bruno et al. found that exosomes from human BM-MSCs inhibit the growth and survival of three different human tumor cell lines. Similar results were observed in NOD/SCID mouse models [120]. Furthermore, exosomes derived from MSCs overexpressing the TRAIL gene-induced apoptosis in a range of cancer cell lines. In another study, mouse BMMSC-derived exosomes were found to suppress tumor progression and angiogenesis in the mouse breast cancer cell line 4T1 by downregulating VEGF expression in vitro and in vivo via shuttling miR-16, which is a known effector of VEGF enriched in MSC-derived exosomes [121].

A different study showed that exosomes derived from menstrual stem cells suppress the secretion of pro-angiogenic factors in prostate tumor cell line PC3 in a reactive oxygen species-dependent manner and inhibit prostate tumor angiogenesis in PC3-bearing NOD SCID gamma mice [122]. Exosomes derived from BMMSCs inhibit cell cycle progression and induce apoptosis in HepG2 cells. Ko et al. showed that AD-MSC-derived exosomes in rat N1S1 cells, an orthotopic HCC model, can promote NKT cell antitumor responses in rats, thereby facilitating hepatocellular carcinoma (HCC) suppression and low-grade tumor differentiation [123]. 

In addition to modulating tumor development, MSCs-derived exosomes have been shown to influence tumor chemosensitivity. Exosomes from human umbilical cord MSCs significantly induce the resistance of gastric cancer cells to 5-fluorouracil in a BALB/c nu/nu mice subcutaneous xenograft tumor model by antagonizing 5-fluorouracil-induced apoptosis and enhancing the expression of multidrug resistance-associated proteins [124]. In another study, exosomes from anti-miR-9-transfected BMMSCs delivered anti-miR-9 into temozolomide-resistant glioblastoma multiforme cells and reversed their chemoresistance by affecting the expression of the multidrug transporter P-glycoprotein [125]. Lou et al. demonstrated that exosomes from miR-122-modified AD-MSC (122-Exo) can mediate miR-122 transfer between AD-MSCs and HCC cells, thereby enhancing cell sensitivity to chemotherapeutic agents by regulating miR-122-target gene expression in HCC cells [105]. The use of MSC-derived exosomes in cancer therapy must be conducted with caution because their role in tumor growth remains elusive. At the same time, their role in tumor suppression also establishes them as promising candidates in cell-free therapy for cancer. A better understanding of the mechanisms involved in regulating MSC-derived exosomes is important to determine their true role in cancer progression and use as a possible therapeutic agent in cancer treatment. 

### 4.6. MSC Derived Exosomes in Lung Diseases

Several studies have shown the efficacy of MSC-derived exosomes as immunosuppressive, anti-inflammatory agents in lung disease [126,127,128,129,130]. The potential of exosomes from BMMSC has also been shown by Khatri et al. in influenza virus-induced acute lung injury in a pig model. The exosomes express cyclooxygenase (COX)-2 mRNA, the enzyme that induces prostaglandin E2 (PGE2) synthesis, which in turn reprogram proinflammatory monocyte-macrophages (M1) to the anti-inflammatory (M2) type. Additionally, MSC exosomes interact with immune cells and cause the production of transforming growth factor (TGF)β and T-regulatory cells (Tregs). Tregs cause a decrease in the haemagglutination activity of influenza viruses and virus replication [131]. Similarly, Yi et al. also reported the inhibition of Serum amyloid A3 by exosomal miR-30b-3p from bone marrow [132]. Furthermore, the authors showed increased cell proliferation and reduction in the apoptosis in type II alveolar epithelial cells of lungs after BMMSC exosome treatment. The role of MSC exosome miRs was also demonstrated by Wei et al. In a lung ischemia/reperfusion injury murine model, the authors showed a reduction of edema and dysfunction in lungs by miR-21-5p [133]. The miR-21-5p further decreases the level of proinflammatory cytokines in the lungs. Heat shock proteins (hsp-90) present in MSC exosomes also play a role in *E. coli*-induced acute lung injury, as demonstrated by Varkouhi et al. [134]. Exosomes derived from MSC in the human umbilical cord are rich in hsp-90, causing a reduced alveolar protein leak and reduction in alveolar TNFα concentrations. Additionally, some reports showed the antimicrobial effect of bone marrow MSC exosomes in *E. coli*-induced acute lung injury [135,136].

#### MSC Derived Exosomes in COVID-19

COVID-19 is the most recent pandemic lung disease to shake the world with its highly infectious and deadly nature. Several clinical trials have employed MSCs and their exosomes against the pathophysiology of COVID-19, showing astonishing outcomes, including the alleviation of symptoms, faster recovery, and phenomenal regeneration [137,138,139]. COVID-19 is caused by SARS-CoV-2 coronavirus, which creates a storm of cytokines in the lungs by invoking major proinflammatory factors, such as CCL-2, CXCL-10, IL-2, IL-6, IL-7, IL-1β, IFN, and TNFα, which attract immune cells and lead to extreme inflammatory conditions [140,141,142,143,144]. It results in deadly damage to the lungs and other organs. In order to combat such a situation, the small size, specificity, and immunosuppressive features of MSC-exosomes can be used. In fact, in a nonrandomized open-label cohort study, Sengupta et al. showed that intravenous administration of BMMSC exosomes improved patients’ clinical status and oxygenation, as evident by the improvements in absolute neutrophil and lymphocyte counts [145]. Additionally, they also showed that these exosomes reduced the level of C-reactive protein, ferritin, and D-dimer in the lungs. However, future randomized controlled trials (RCTs) are needed to determine its therapeutic potential. 

## 5. Exosomes as a Drug Delivery Vehicle

Exosomes are already recognized as novel therapeutic modalities, but these vesicles also possess a natural homing ability and can travel long distances which confirms their suitability as a drug delivery vehicle. Due to their small size and native nature, they are even able to cross physiological barriers. Exosomes are known to be stable in circulation and go undetected by the immune system, thereby aiding their longer viability and existence in the biological system. Some studies have found that exosomes remain intact even when subjected to digestive enzymes, hence sequestering their cargo from degeneration [146]. 

**Figure 4:**
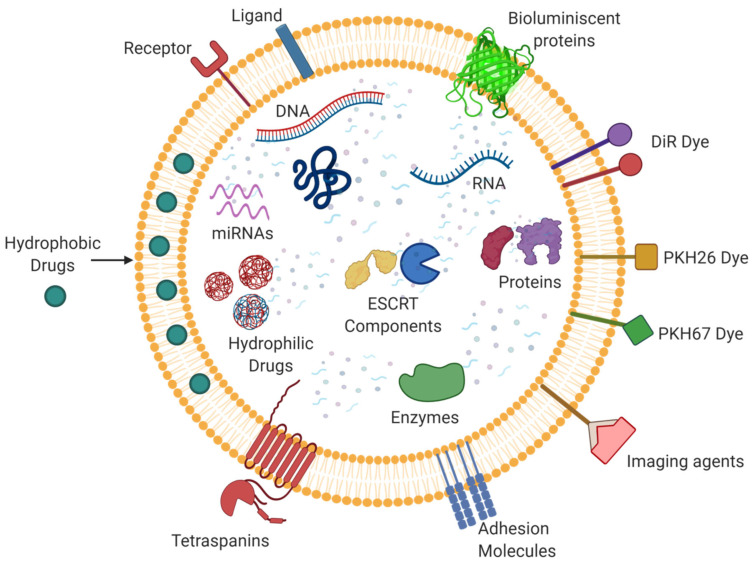
The multifaceted nature of exosomes: Exosomes can carry varied cargo including DNA, RNA, miRNAs, enzymes, proteins, ESCRT components, etc. They can also serve as drug delivery systems through their ability to carry and transfer a wide range of molecules, such as hydrophobic and hydrophilic drugs. Multiple molecules, including dyes, imaging agents, etc. can also be linked to exosomes for their visualization and characterization.

Their current competitor as a drug delivery system is the liposome. Liposomes are synthetically made structures with a lipid bilayer and can encapsulate both hydrophilic and hydrophobic moieties. However, the shift in focus from liposomes to exosomes is due to the former’s synthetic nature and laborious production protocols [147]. So, this kind of extensive chemical treatment can be circumvented by the application of naturally released exosomes. However, exosomes have some lacunae to act as an effective drug delivery system, especially hydrophilic macromolecules [148]. The exosomal membrane resembles the cell membrane that is lipophilic in nature, and since it has a hydrophilic core, it holds the ability to carry water-soluble drugs (Figure 4). Liang et al. engineered HEK-293T cell line-derived exosomes to contain 5-Fluorouracil and miR-21i for reversal of drug resistance aiding chemotherapy in colorectal carcinoma [149]. Considering the simplicity in its structure and comprehension in its behavior, we realize the potential of exosomes to be employed as a drug delivery vehicle by manipulating its cargo as they are naturally derived and hence will not be considered foreign to the body. Therefore, they are conferred with the ability to last longer in the system and enhance the efficiency of therapy. They are highly specific in targeting and can therefore be called a “smart” vehicle for drug delivery. Research in the field of exosomes/extracellular vesicles is emerging, and extensive research is needed for future therapeutic applications. It also warrants the attention of researchers worldwide owing to its ability to transfer therapeutics to recipient cells via an endogenous uptake mechanism; therefore, the exosome vesicle is a promising candidate [150]. Although liposomes are a well-standardized drug delivery system, we strongly believe that the naturally secreted exosomes will stand as a promising therapeutic candidate in a wide spectrum of human diseases with incoming decades.

Currently, most exosomes employed as drug delivery vehicles are derived from plant sources. However, since the potential of exosomes as a drug delivery vehicle and the renowned capabilities of mesenchymal stem cells (MSCs) are already established, we can infer that exosomes derived from MSCs hold great potential for becoming an all-in-one smart therapeutic agent and drug delivery vehicle. Exosomes derived from MSCs display immense immunoregulatory and anti-inflammatory properties [151,152]. WJ-MSC-derived exosomes loaded with miR-146a represent appreciable therapeutic efficiency in inflammatory disorders by promoting macrophage polarization, as shown by Song et al. MSCs and their derivatives can travel to the site of tumors [153]. Sharif et al. showed that exosomes derived from WJ-MSC were able to assist in miRNA replacement therapy in glioblastoma multiforme cancerous cells [154]. Exosomes derived from MSCs have also proven instrumental in many other disease models. Tian et al. modified the surface of BM-MSC-derived exosomes by conjugating a peptide for targeted delivery to the lesions in the ischemic brain [155]. Hence, if we employ exosomes derived from MSCs as drug delivery vehicles, they can serve dual protective functions, i.e., aid in healing by manifesting the curative properties of its own, thereby creating a microenvironment that promotes healing, and functioning as a targeted vehicle for the delivery of respective drugs.

## 6. Limitations and Leads for the Future

Exosomes are potentially future avenues in therapeutics and drug delivery systems. Although exosomes have attracted much clinical interest recently owing to their noteworthy properties, certain aspects need critical assessment for the pharmaceuticalization of exosomes and their use as drug delivery vehicles. First, it is imperative to cautiously study and scrutinize the source cell for the derivation of exosomes. The structural composition and surface markers of exosomes are essential for their physiological activity [156]. These properties are a characteristic of the source cell of exosomes and may be considered a factor while specifying the motive of therapy. The cargo of exosomes depends upon the cell’s physiological state, and some reports have shown that exosomes derived from cancer cells, for e.g., may exhibit progressive tumor activities or modulation of the immune system in a harmful manner. Exosomes from any source can also encapsulate pre-existing infection in the source cell. Therefore, differential verification of cargo, purity of exosomes, and their downstream effector functions need to be assessed critically before being processed for therapeutic uses. 

Many details about the characteristics, properties, and cargo of exosomes are also available through online sources and databases like ExoCarta (http://exocarta.org/exosome_markers_new accessed on 26 July 2021), ExoBCD (https://exobcd.liumwei.org/ accessed on 26 July 2021), Vesiclepedia (http://microvesicles.org/ accessed on 26 July 2021), etc. which provide bioinformatics-based information in a comprehensive and detailed manner. These databases can be referred to as a starting point of any exploratory project regarding novel research in EV-Science. However, even these sources must be verified and updated regularly.

It is also imperative to thoroughly understand the physiology and mechanism of action of exosomes for their wholesome utilization and application. Another barrier to using these nanovesicles in clinical application is always the risk of off-target functioning. In order to combat this, specificity can be improved by introducing targeting peptides or ligands on the exosome surface by conjugation via techniques like click chemistry to improve the exosome binding on the target cell [157]. Apart from that, standardization of basic protocols is necessary to improve the yield and large-scale production of exosomes to render them competitive for commercial drug manufacturing. Insight into the biogenesis of exosomes might provide an answer to increasing the yield of exosomes. However, certain reports have suggested that preconditioning the MSCs in hypoxia may improve the yield of exosomes significantly [158,159,160,161]. 

Another approach to derive an improved yield of EVs is by using bioreactors as = culture systems. In this system, a hollow fibrous bioreactor creates a huge surface area for cell attachment, while the media is automatically and continually added; this allows long term maintenance of the culture without passaging and simultaneously producing large volumes of conditioned culture media for EV extraction [162]. Del Piccolo et al. showed EV release from CHO cells using vesiculation buffer [163]. The authors showed that rinsing the cells first with hypotonic buffer and further with hypertonic buffer, stressed them sufficiently that the release of vesicles into the solution increased [163]. However, the exact mechanism is not understood. Similarly, increasing the yield of exosomes by extrusion through a polycarbonate membrane with pore sizes of 400, 200, and 100 nm has been suggested by Emam et al. [164]. In addition to the approaches above, induction of the release of membrane vesicles from the surface of the PC3 cell line by Cytochalasin B, shown by Gomzikova et al. as Cytochalasin B, destabilizes cytoskeletal and membranous interactivity [165,166]. 

**Figure 5:**
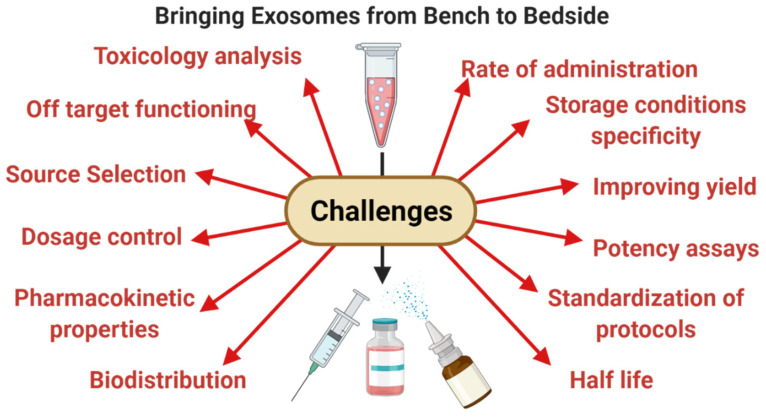
The challenges of bringing exosomes from bench to bedside: Even after the multifactorial facets of MSC derived exosomes, there are still many challenges in their ultimate translation into a product; these may include low yield, uncertainty in yield, potency, half-life, biodistribution, source selection, risk of off target functioning, etc.

Furthermore, it is also essential to define the storage conditions and handling procedures in order to bring exosomes to the clinic. In-depth studies must be devised to gain insights into the toxicology data, pharmacokinetic properties of exosomes, potency, biodistribution, dosage control, and the best route of administration as this will assist in the composing suitable formulations of exosomes and the eventual commercialization of exosomes as a therapeutic aid and drug delivery vehicle (Figure 5).

## 7. Conclusions

In summary, we can infer that exosomes distinguish themselves as an eminent novel modality for therapeutics and drug delivery. Their small size and undemanding operational protocols highlight them as a practical candidate for an off-the-shelf remedial approach; this has directly implies the commercialization and mass manufacturing of these nano-vesicles for standardization in isolation and characterization techniques. MSCs as a source of exosomes have gained a lot of research interest for their immunomodulatory nature and remedial potential in several diseases. They hence can be considered as an ideal source for the derivation of exosomes and therapeutic applications. However, for their complete utility, applicability, and pharmaceuticalization, mechanistic insights must be gained about their production and mechanism of action, and studies for the standardization of protocols for exosome handling are warranted.

## Figures and Tables

**Table 1:** Comparison between MSCs and MSC derived Exosomes.

MSCs	MSC Exosomes
Low stability	High Stability
High immunogenicity	Low immunogenicity
Cannot cross blood brain barrier	Can easily cross blood brain barrier
High-cost storage	Low-cost storage
Can-not be readily used as off-the-shelf	Potential for off-the-shelf availability

**Table 2:** A summary of the mechanism of action of MSC-derived exosomes against different diseases.

Disease	Cell Source	Exosome Content	Mechanism of Action	Reference
Myocardial ischemia/reperfusion injury	hESC derived MSC	Not given	Out of the complex mixture of nutrients, growth factors, microvesicles etc. in the conditioned media, exosomes are specifically responsible for tissue repair and cardioprotective effects in case of ischemia/reperfusion injury	[68]
Acute Myocardial Infarction	hESC derived MSC	Peroxiredoxins and glutathione S-transferase, enzymatically active CD73	Increased levels of ATP and NADH, decreased oxidative stress, increased phosphorylated-Akt and phosphorylated-GSK-3β	[69]
Myocardial Infarction	hBMMSC	Sonic hedgehog, PDGFR	Increased angiogenesis, HIF-1 alpha activation	[70]
Ischemic heart	Mice BMMSC	miR22	Targeting the methyl CpG binding protein 2 (Mecp2)	[71]
Myocardial Infarction	MSC overexpressing GATA-4	miR-19a, miR-451, miR-221, IGF-1	Anti-apoptotic effect, reduction in PTEN and BIM expression, Akt/ERK signalling pathway	[72]
Myocardial Infarction	Human Endometrium-derived MSC (EnMSC)	miR-21	PTEN/Akt pathway	[73]
Acute Myocardial Infarction	Atorvastatin treated MSC	lncRNA H19	Increased angiogenesis, inhibited the elevation of IL-6 and TNF-α	[74]
H9C2 cardiomyocyte	Mice BMMSC	miR-144	PTEN/Akt pathway	[75]
Myocardial Infarction	Mice BMMSC	miR-210	Reduce apoptosis of cardiomyocytes, AIFM3/p53 and PI3K/Akt signaling pathways	[76]
Stroke	Rat BMMSC	miR-133b	Enhanced neurological recovery, stimulated neurogenesis and angiogenesis	[77]
Stroke	hBMMSC	Not given	Stimulated neurogenesis and angiogenesis	[78]
Parkinson’s disease	Human dental pulp stem cells	Not given	Suppressed 6-OHDA-induced apoptosis in dopaminergic neurons	[82]
Age-related macular degeneration	Retinal pigment epithelial cells	αB crystallin	Inhibition of caspase 3 and PARP activation	[84]
Parkinson’s disease	Mouse macrophage cell line	Catalase	Reduced Oxidative stress	[85]
Alzheimer’s disease	hADMSC	Neprilysin	β-amyloid peptide degradation	[86]
SH-SY 5Y human neuroblastoma cells	murineADMSC	Not given	Reduction of neuronal apoptosis	[88]
Amyotrophic lateral sclerosis	murineADMSC	miR21, miR222, miRlet7a	Apoptosis-inhibiting pathway, cell cycle progression	[87]
Acute kidney injury	hBMMSC	IGF-1R	Increased proximal renal tubular epithelial cell proliferation	[92]
Acute kidney injury	hBMMSC	mRNA	Induced de-differentiation of mature cells, triggered proliferation	[93]
Acute kidney injury	hMSC	Not given	Upregulated anti-apoptotic genes Bcl-xL, Bcl2 and BIRC8 in tubular epithelial cells	[96]
Renal proximal tubular epithelial cells	hBMMSC	let7- a, miR-148b-3p, 375, 410, 451, 485-3p, 495, 522, 548c-3p, 548c-5p, 561, and 886-3p	Downregulation of apoptotic genes, SHC1 mediated inhibition of EGFR-Ras-ERK pathway	[97]
Renal ischemia/reperfusion injury	hWJMSC	Not given	Supress expression of NOX2, ROS level reduction	[98]
Renal ischemia/reperfusion injury	hWJMSC	miR-15a, miR-15b and miR-16	Downregulation of CX3CL1	[99]
Chronic kidney disease	hCBMSC	Not given	Increase in TGF-β1 and IL-10 levels, decrease in TNF-α levels	[100]
Chronic liver fibrosis	murineBMMSC	Not given	Inhibition of hepatocellular apoptosis, inhibition of proliferation of LX-2	[98]
Liver fibrosis	hUCMSC	Not given	Inhibition of EMT, inactivation of the TGF-β1/Smad signalling	[103]
Liver fibrosis	chorionic plate-derived mesenchymal stem cells (CP-MSCs)	miR-125b	Downregulation of hedgehog signaling	[104]
Liver fibrosis	ADMSC	miR-122	proliferation and collagen maturation of HSCs	[106]
Liver Injury	hESC-derived HuES9.E1 MSC	Not given	Up regulation of PCNA and cyclin D1, inhibition of the APAP- and H_2_O_2_-induced hepatocytes apoptosis	[107]
Acute liver Injury	hUC-MSC	miR-455-3p	PI3K signaling, inhibition of IL-6-related signaling pathways, suppress monocyte/macrophage activation	[108]
Acute liver failure	miceADMSC	miR-17	Suppress NLRP3 inflammasome activation	[109]
Prostate cancer	hADMSC	miR-145	Inhibit cell proliferation, inducing apoptosis	[113]
Multiple Myeloma	hBMMSC	miR-15a	Inhibited the growth of MM cells	[114]
Renal cancer	hWJMSC	HGF mRNA	Activation of AKT and ERK1/2 signaling pathways, reduction of HGF expression	[115]
Human gastric carcinoma	hBMMSC	VEGF	Inhibition of ERK1/2 activation	[116]
Nasopharyngeal carcinoma	hBMMSC	FGF19	FGF19-FGFR4-dependent ERK signaling	[117]
Breast cancer cell line (MCF-7)	hSDMSC	miR-21, miR-34a, PDGFR-β, TIMP-1, and TIMP-2, sphingomyelin, lactic acid, glutamic acid	Inhibited cell death, anti-proliferative effect	[118]
Osteosarcoma (MG63) and gastric cancer (SGC7901) cells	hBMMSC	Not given	Hedgehog signaling pathway	[120]
Mouse breast cancer cell line (4T1)	miceBMMSC	miR-16	Inhibition of tumor growth and angiogenesis, reduces the VEGF expression	[121]
Prostate Adenocarcinoma PC3	Menstrual MSC	Not given	Reduction in VEGF secretion and NF-κB activity, lower ROS	[122]
Hepatocellular carcinoma	ratADMSC	*β*-catenin	Promoted NKT-cell antitumor responses, low-grade tumor differentiation	[123]
Glioblastoma multiforme	hBMMSC	anti-miR-9	Reduced miR-9, cell surface P-gp	[125]
Hepatocellular carcinoma	miR-122-modified AD-MSC	miR-122	Enhancing cell sensitivity to chemotherapeutic agents	[106]
Acute lung injury by Influenza virus	Swine BMMSC	Cyclooxygenase (COX)-2 mRNA, Indoleamine 2,3-dioxygenase (IDO)	Reduce Haemagglutination activity of influenza viruses, virus replication, decrease in proinflammatory cytokine production	[131]
LPS induced Acute lung injury	BMMSC overexpressing miR-30b-3p	miR-30b-3p	Decreased SAA3 level, increased cell proliferation, reduce apoptosis	[132]
Lung Ischemia/Reperfusion injury	Murine BMMSC	miR-21-5p	Reduced lung edema and dysfunction, M1 polarization of alveolar macrophages, increase secretion of HMGB1, IL-8, IL-1β, IL-6, IL-17 and TNF-α	[133]
*E. coli*-induced acute lung injury	hUCMSC	hsp-90	Reduced alveolar protein leak, increased lung mononuclear phagocytes, reduced alveolar tumor necrosis factor alpha concentrations	[134]
*E. coli* induced acute lung injury	hBMMSC	Not given	Decrease in lung protein permeability, increased alveolar fluid clearance, antimicrobial effect	[135]
Acute lung injury due to severe pneumonia (*E. coli* induced)	hBMMSC	Not given	Reduced inflammation, total bacterial load, lung protein permeability, increase monocyte phagocytosis, Restored intracellular ATP levels in injured human ATII cells	[136]
COVID-19	hMSC (UCMSC, BMMSC, ADMSC, dental pulp MSC)	Not given	Inhibit macrophage accumulation and activation, cytokine strome reduction, reduction in CD4^+^ T cells, CD8^+^ T cells	[137]
COVID-19	BMMSC	ExoFlo™	Improved oxygenation, improvements in absolute neutrophil count, C-reactive protein, ferritin, and D-dimer reduction	[145]
